# Interface Tailoring Effect for Heusler Based CPP-GMR with an *L*1_2_-Type Ag_3_Mg Spacer

**DOI:** 10.3390/ma11020219

**Published:** 2018-01-31

**Authors:** Takahide Kubota, Yusuke Ina, Zhenchao Wen, Koki Takanashi

**Affiliations:** 1 Institute for Materials Research, Tohoku University, Sendai 980-8577, Japan; inaxy.com@gmail.com (Y.I.); wen.zhenchao@imr.tohoku.ac.jp (Z.W.); koki@imr.tohoku.ac.jp (K.T.); 2 Center for Spintronics Research Network, Tohoku University, Sendai 980-8577, Japan

**Keywords:** CPP-GMR, heusler alloy, hard disk drive, spintronics, magnetoresistance

## Abstract

Current perpendicular-to-plane (CPP) giant magnetoresistance (GMR) effects are of interest in a possible application of magnetic sensor elements, such as read-head of hard disk drives. To improve the junction performance, the interface tailoring effects were investigated for the Heulser alloy, Co_2_Fe_0.4_Mn_0.6_Si (CFMS), based CPP-GMR junctions with an $L1_{2}$-Ag_3_Mg ordered alloy spacer. Ultra-thin Fe or Mg inserts were utilized for the CFMS/Ag_3_Mg interfaces, and CPP-GMR at low bias current density, *J* and the *J* dependence were evaluated for the junctions. Although, at low bias *J*, MR ratio decreased with increasing the inserts thickness, the device output at high bias *J* exhibited quite weak dependence on the insert thickness. The output voltages of the order of 4 mV were obtained for the junctions regardless of the insert at an optimal bias *J* for each. The critical current density $J_{c}$ was evaluated by the shape of MR curves depending on *J*. $J_{c}$ increased with the insert thicknesses up to 0.45 nm. The enhancement of $J_{c}$ suggests that spin-transfer-torque effect may reduce in the junctions with inserts, which enables a reduction of noise and can be an advantage for device applications.

## 1. Introduction

Current perpendicular to plane (CPP) giant magnetoresistance (GMR) junction is potentially utilized for highly sensitive magnetic sensor applications, e.g., read-head devices for hard disk drives (HDDs) [1,2,3,4]. A merit of CPP-GMR junction is a relatively low areal resistance (RA) of the order of 10^−2^–10^−3^Ω·μm^2^, which is an advantage over tunnel magnetoresistance (TMR) junctions. The low RA in the optimum range applicable to the next generation HDDs with areal recording density of several tera-bit-per-square-inches [3]. Magnetic tunnel junctions (MTJs) with an MgO barrier exhibit a high MR ratio over 100% with RA down to 1 Ω·μm^2^ [5,6]; however, the MR ratio decreases for a lower RA region because of the ultra-thin tunneling barrier. The reduction of the MR with the reduced barrier thickness is unavoidable from the principle of coherent tunneling [7,8]. Thus, we can say that an advantage of CPP-GMR is the low RA compared to MgO-MTJs, which is currently used for HDD read-heads, and the development of CPP-GMR is necessary for futre HDD tecnology. According to the theoretical model proposed by Valet and Fert in 1993 [9], spin asymmetry coefficients crucially affect the resistance change of CPP-GMR junctions, which are defined by magnetic material as well as the interface between a non-magnetic spacer and the magnetic layer. For realization of high output for the CPP-GMR junctions, magnetic materials with high-spin polarization are essential. One of the developed magnetic material classes for CPP-GMR junctions is half-metallic cobalt (Co)-based Heusler alloys for some of which the theoretically predicted spin polarization is 100% [10]. In experiments for CPP-GMR, a pioneer work has been reported for junctions using Co_2_MnSi Heusler alloy and a Cr spacer, in which relatively large resistance change was observed [11]. The next trigger was given by a Ag spacer for the Co_2_-Heusler junctions, in which MR ratio and the areal resistance change (ΔRA) reached to about 30% and 10 mΩ·μm^2^, respectively, at room temperature [12,13]. To date, several Heusler alloys are known as half-metallic and have been utilized for CPP-GMR junctions, e.g., Co_2_(Al-Si) [13,14], Co_2_MnGe [15,16], Co_2_(Fe-Mn)Si [17,18], and Co_2_(Ga-Ge) [19]. Selection of the spacer material is another crucial factor for CPP-GMR from a viewpoint of band-dispersion-matching to the Heusle alloy layers [20,21,22,23], for the purpose of which various non-magnetic materials have also been investigated experimentally, e.g., Cr, Ag, Cu, non-magnetic Heusler alloys [20,24,25], B2-type NiAl [26], noble-metal(Cu or Ag)-Zn alloys [27,28], conductive oxide [29], and so on.

**Figure 1:**
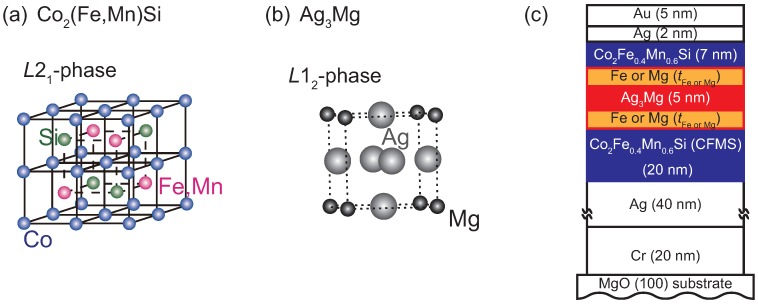
Schematic illustrations of crystal structures for (**a**) Co_2_(Fe,Mn)Si (CFMS) full Heusler alloy in the $L2_{1}$ phase; (**b**) Ag_3_Mg alloy in the $L1_{2}$ phase, and (**c**) stacking structure of the layered films for current perpendiculr to plane (CPP) giant magnetoresistance (GMR) junctions.

Among the numerous materials we have focused on a Co_2_Fe_0.4_Mn_0.6_Si (CFMS) alloy as a half-metallic material, and an $L1_{2}$ Ag_3_Mg alloy as a spacer, for which schematic crystal structures are shown in Figure 1a,b, respectively. CFMS is a known half-metallic material on the bases of both theoretical and experimental studies [18,30,31]. One of the merits is a high exchange stiffness at the interface which is important for realizing large CPP-GMR at room temperature [32,33]. The $L1_{2}$ Ag_3_Mg is an ordered alloy spacer material for which good band-dispersion-matching to CFMS has been predicted [34], and large CPP-GMR have experimentally demonstrated for CFMS/$L1_{2}$ Ag_3_Mg/CFMS junctions, which is an advantage over the Ag spacer junctions [34,35,36].

In this work, the interface tailoring effect is investigated for the CFMS/$L1_{2}$ Ag_3_Mg/CFMS junctions to improve the output performance. According to literatures [37,38], ultra-thin inserts at Heusler layer/spacer interfaces drastically modified the CPP-GMR properties. For the insert materials, Fe and Mg have been selected. The Fe insert is interested in enhancing the exchange stiffness at the interface [33,38], and the Mg insert is interesting because the electronic state consists of sp-components only [39], which may result in a filtering effect for the highly spin polarized electrons of Heusler alloys with sp-like symmetry.

## 2. Materials and Methods

Layered films were deposited onto MgO (100) single crystalline substrates by using an ultra-high vacuum magnetron sputtering system for which base pressure of the vacuum chamber was less than 1 × 10^−7^ Pa. For the sputtering power sources, radio frequency source was used for the Ag target, and direct current source was used for other targets. The stacking structure of samples is as follows and shown in Figure 1c: Cr (20 nm) | Ag (40 nm) | CFMS (20 nm) | Fe or Mg ($t_{\mathrm{FeorMg}}$) | Ag_3_Mg (5 nm) | Fe or Mg ($t_{\mathrm{FeorMg}}$) | CFMS (7 nm) | Ag (2 nm) | Au (5 nm), from bottom to top. The film compositions of the CFMS and Ag_3_Mg layeres were Co_47_Fe_13_Mn_15_Si_25_ (at.%) and Ag_78_Mg_22_ (at.%), respectively. The thicknesses for the inserts, $t_{\mathrm{FeorMg}}$, ranged from 0 to 0.6 nm in 0.15 nm increments. The surface of the MgO substrates was flushed at 650 °C by using an infrared heater equipped inside the ultra-high vacuum chamber. All layeres were deposited at room temperature and *in situ* post-annealing was carried out at 650 and 550 °C after the deposition of the Cr and the upper CFMS layer, respectively. Crystalline property of the films was examined using reflection high energy electron diffraction, RHEED, patterns for the surface of the upper CFMS electrode. The layered films were patterned into a pillar shape with sub-micrometer-scale using electron-beam lithography and argon ion dry etching technique. Designed pillar sizes ranged from 50 × 100 nm^2^ to 400 × 800 nm^2^ with a rectangular shape. And actual pillar sizes were estimated using the same method as that in our previous studies [34,40], which ranged from 40 × 80 nm^2^ to 390 × 780 nm^2^, approximately. The RA values of junctions were estimated using resistance at the parallel magnetization configuration, $R_{p}$, as a function of the inverse junction area, 1/A, where A is in the unit of μm^2^. The measuerments of $R_{p}$ were carried out for all sizes of junctions. The detail for the procedure of RA estimation was described in a previous paper of ours [36]. CPP-GMR effects were measured by four-probe method at room temperature. In this paper CPP-GMR effects were characterized by two aspects: CPP-GMR effects at low bias current density, *J* and the bias *J* dependence of CPP-GMR. For the measurements at low *J*, the current was fixed so that the applied bias voltage was about 1 mV at a parallel magnetization configuration, and the magnetic field was swept along a long axis of the pillar shape which is an easy magnetization axis. Regarding the value of MR ratio, two definitions are used: The observed MR ratio, MR_obs_, and the intrinsic MR ratio, MR_int_, for which the parasitic resistance, $R_{\mathrm{para}}$ was subtracted from $R_{P(\mathrm{AP})}$, where $R_{\mathrm{AP}}$ represents the junction resistance at antiparallel magnetization configuration. Here, $R_{\mathrm{para}}$ originates from the resistance of the lead electrodes for the junction. The values are defined by the following equations;

(1) $${\mathrm{MR}}_{\mathrm{obs}}=\frac{R_{\mathrm{AP}}-R_{P}}{R_{P}}\times 100\left(\%\right),$$

(2) $${\mathrm{MR}}_{\mathrm{int}}=\frac{R_{\mathrm{AP}}-R_{P}}{R_{P}-R_{\mathrm{para}}}\times 100\left(\%\right),$$

And ΔRA is also defined as follows;

(3) $$\Delta RA=\left(R_{\mathrm{AP}}-R_{P}\right)A=RA\times {\mathrm{MR}}_{\mathrm{int}},$$

For the *J* dependence, GMR curves were measured at several values of *J* within the range of about 10^8^ A/cm_2_, and the magnetic field was swept along a short axis of the pillar shape, which is a hard magnetization axis direction.

## 3. Results and Discussion

**Figure 2:**
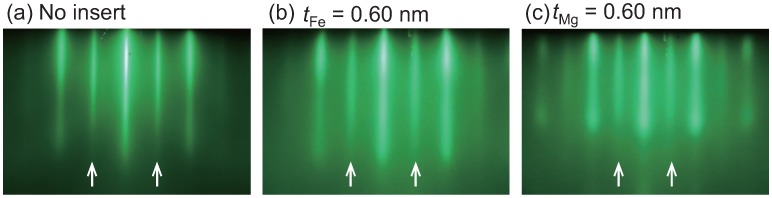
Reflection high-energy electron diffraction (RHEED) images for the surface of the upper CFMS layer for azimuth of CFMS <110>. Layered films with (**a**) no insert; (**b**) Fe inserts of 0.60 nm, and (**c**) Mg inserts of 0.60 nm. The arrows indicate the superlattice streaks from the $L2_{1}$ phase of CFMS.

RHEED images are shown in Figure 2 for the surface of the upper CFMS layer in the layered films for CFMS<110> azimuth. The thickness of the inserts is 0.6 nm for Fe (Figure 2b) or Mg (Figure 2c), which is the maximum thickness of the inserts among the samples. Regardless of the inserts, streaks are clearly observed including the superlattice streaks from the $L2_{1}$ phase of the CFMS layer which are pointed by arrows. RHEED images for another azimuth of CFMS<100> also showed streak patterns including the fundamental diffractions only (not shown here). RHEED images for other insert-thicknesses samples also showed similar features. Thus, it is confirmed that the epitaxial growth and the $L2_{1}$ phase in the CFMS electrode are maintained for all samples including the samples with Fe or Mg inserts.

**Figure 3:**
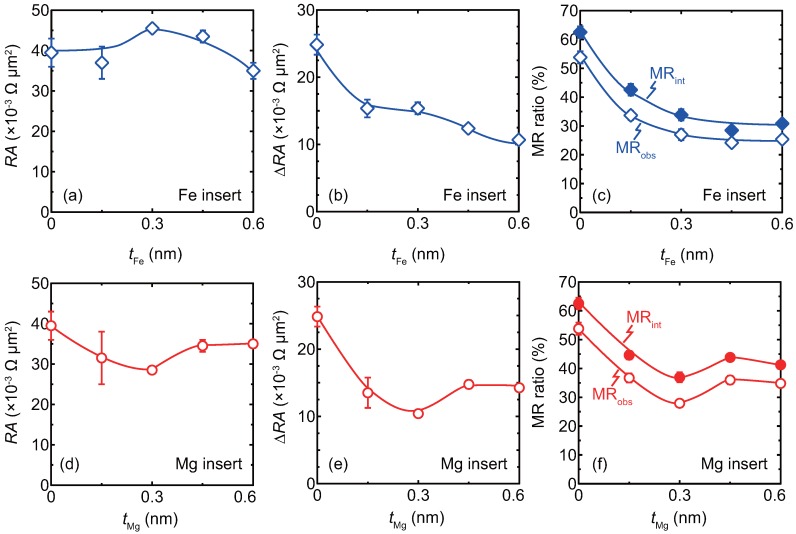
A summary of the insert thickness dependence of CPP-GMR at low bias current density for junctions with (**a**–**c**) Fe inserts and (**d**–**f**) Mg insert. (**a**,**d**) RA, (**b**,**e**) ΔRA, and (**c**,**f**) MR ratios. Definitions of the observed MR ratio, MR_obs_, the intrinsic MR ratio, MR_int_, and ΔRA are shown in Equation (1), Equation (2), and Equation (3), respectively.

Summaries of RA, ΔRA, and MR ratios at low bias *J* are shown in Figure 3a,d, Figure 3b,e and Figure 3c,f, respectively. Figure 3a–f indicate the inserts thickness dependence of the junctions with the Fe inserts and the Mg inserts, respectively. The error bars represent the standard deviation estimated from the measured junctions.

Regarding the junctions with Fe inserts, the RA value slightly increases with $t_{\mathrm{Fe}}$ of up to 0.45 nm and decreases for $t_{\mathrm{Fe}}$ of 0.60 nm. Both ΔRA and MR ratios monotonically decrease with increasing $t_{\mathrm{Fe}}$. Although the reason for the RA dependence is unclear, a change of band-matching is a possible factor caused by the inserts [23,37]. On the other hand, regarding the junctions with Mg inserts, RA, ΔRA and MR ratios exhibit smaller values than those for junctions with no insert . For the junctions with Mg inserts, ΔRA and MR ratios show non-monotonic decrease, which is different from junctions with Fe inserts . The difference between the Fe and Mg inserts is discussed as follows: Firstly the Fe inserts possibly formed an alloy with the CFMS layer by annealing at 550 °C. Assuming that the interfaces became Fe-rich CFMS, the spin polarization was smaller than that of the original CFMS composition, which was reported by Sakuraba et al. [18]. Even in case that the Fe layers remained at the interfaces, the spin polarization would be smaller than that of the original CFMS/Ag_3_Mg interfaces, which is similar with a work done by Jung et al. [38]. On the other hand for the Mg inserts, the Mg possibly remained as Mg-layeres at the CFMS/Ag_3_Mg interfaces, because Mg atoms are less soluble with the CFMS layers, which was discussed in a previous paper of ours [36]. The ΔRA and the MR ratios decrease due to the reduced interface spin asymmetry at the Mg interfaces. Concerning the non-monotonic decrease for the ΔRA and the MR ratios, a conduction channel through the quantum states in the ultra-thin Mg layer is a possible factor [39].

**Figure 4:**
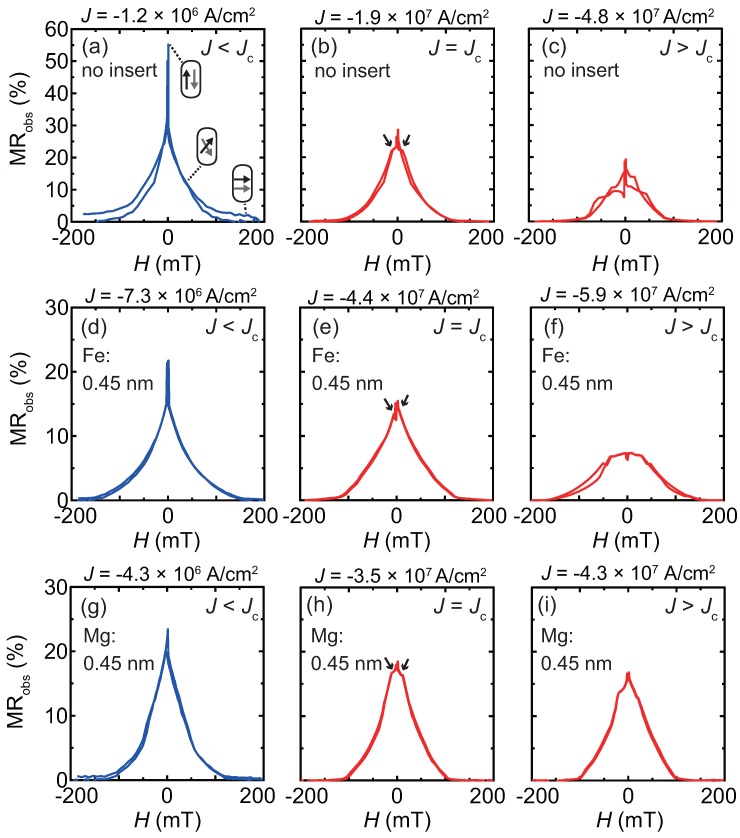
Bias current density, *J* dependence of MR curves. Junctions with (**a**–**c**) no insert; (**d**–**f**) Fe inserts, and (**g**–**i**) Mg inserts. The thickness of the inserts is 0.45 nm.

Figure 4 shows typical MR curves for the investigation of bias *J* dependence, the shapes of which are explained as follows for the low bias region, $J<J_{c}$: Firstly, magnetizations of the CFMS layers are coupled with antiferromagnetically through the dipolar field from the pillar edges, thus the MR ratios exhibit a maximum value at the zero magnetic field, H=0. With increasing *H*, magnetizations gradually rotate, the behavior of which is similar with that of the edges of scissors schematically illustrated by arrows in Figure 4a. By applying sufficiently large *H*, e.g., 200 mT, magnetizations aligned in parallel configuration and the junction resistance becomes a minimum. A similar shape of MR curve was reported in the literature for a Heusler alloy based CPP-GMR with antiferromagentic coupling [41].

From the shape of MR curves, the critical current density, $J_{c}$, is defined at the point where small shoulders start to appear in the MR curve. The shoulders are pointed by arrows in Figure 4b, Figure 4e and Figure 4h for junctions with no insert, 0.45 nm-thick Fe inserts, and 0.45 nm-thick Mg insert, respectively. For $J>J_{c}$, the shapes of MR curves collapse because of the unstable antiparallel configuration of the magnetization which were fluctuated by spin-transfer torque (the curves are shown in Figure 4c, Figure 4f and Figure 4i for no insert, the Fe inserts, and Mg inserts junctions, respectively).

**Figure 5:**
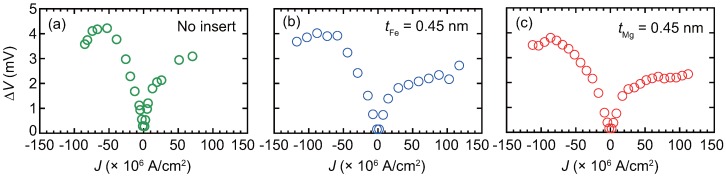
Bias current density, *J*, dependence of the output voltage, ΔV for the junctions with (**a**) no insert; (**b**) Fe inserts, and (**c**) Mg inserts. The thickness of the inserts is 0.45 nm for (**b**,**c**).

The *J* dependence of the output voltage (ΔV) is shown in Figure 5 for a junction with no insert and junctions with 0.45 nm-thick Fe or Mg inserts. Here ΔV is defined by the following equation:

(4) $$\Delta V\equiv \Delta R\times |I_{\mathrm{bias}}|=\left(R_{\max}-R_{H=200\mathrm{mT}}\right)\times \left|I_{\mathrm{bias}}\right|,$$

where $R_{\max}$, $R_{H=200\mathrm{mT}}$, and $I_{\mathrm{bias}}$ represent the maximum resistance in the MR curve, resistance at H=200 mT, and the bias current for the measured MR curve, respectively. The positive bias is defined by the electron flow from the bottom to top. The ΔR for each data point was obtained by sweeping an MR curve at a bias *J*, for which examples are shown in Figure 4. ΔV shows asymmetric dependence on *J*, because the volume of the upper CFMS layer is smaller than that of the bottom: Considering the effect of spin-transfer-torque (STT), the parallel magnetization configuration is stabilized by the electron flow for the positive bias, while the antiparallel configuration is stabilized for the negative bias. ΔV exhibits a maximum value at *J* at around −90–−50 $\times 10^{6}$ A/cm_2_, which is defined as $\Delta V_{\max}$.

**Figure 6:**
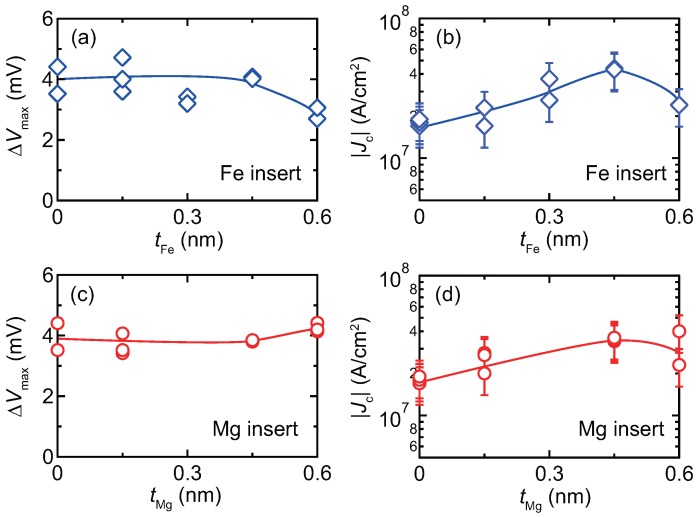
Summaries of (**a**,**c**) the maximum output voltage, $\Delta V_{\max}$, and (**b**,**d**) the critical current density $J_{c}$ as a function of the insert thickness. (**a**,**b**) Fe insert, (**c**,**d**) Mg insert.

Summaries of $\Delta V_{\max}$ and $|J_{c}|$ as a function of the insert thickness, $t_{\mathrm{FeorMg}}$ are shown in Figure 6. For the Fe insert junctions, $\Delta V_{\max}$ of the order of 4 mV is obtained for $t_{\mathrm{Fe}}$ in the range of 0.45 nm, and the value drops to about 3 mV for $t_{\mathrm{Fe}}$ = 0.60 nm. For the Mg insert junctions, $\Delta V_{\max}$ is almost independent of $t_{\mathrm{Mg}}$ and the values are about 4 mV. Regarding the dependence of $J_{c}$, the values increase with the insert thickness of up to 0.45 nm for the Fe insert junctions and decreases at 0.60 nm, which is similar to the dependence for the Mg insert junctions. The increase of $J_{c}$ can be qualitatively explained that the STT effect reduced because of the reduced spin polarization which correlates with MR ratio. In addition, for the case of the Fe insert junctions, enhancement of Gilbert damping constant is another possible factor [42,43], which enhances torque acting towards opposite direction to that of the STT [44]. Although MR ratio was degraded at low bias *J* by the inserts, ΔV almost unchanged because of the balance between the spin polarization and the STT effect. Considering CPP-GMR for read head application of HDDs, the noise caused by STT is an issue to be suppressed [3]. From the bias *J* dependence for the present junctions, it is suggested for the junction with inserts that the ΔV can be maintained under a condition with smaller STT effect. Such a device feature is favorable for the read head or similar sensor applications.

## 4. Conclusions

Interface tailoring effect was investigated for the CPP-GMR junctions including the CFMS/Ag_3_Mg/CFMS structure with the ultra-thin Fe or Mg inserts. Epitaxially grown layered structures were successfully fabricated including the samples with the Fe or Mg inserts of the thickness up to 0.60 nm. At low bias *J*, MR ratio decreased with the insert thickness. On the other hand, the junctions output ΔV was almost unchanged by the inserts, which was of the order of 4 mV. These trends were similar between the junctions using Fe and Mg inserts. The inserts possibly suppressed the STT effect, resulting in the relatively large ΔV at the large bias *J*. The suppressed STT is considered as a merit of the insert for CPP-GMR junctions for device applications.

## References

[1] Takagishi M., Koi K., Yoshikawa M., Funayama T., Iwasaki H., Sahashi M. (2002). The applicability of CPP-GMR heads for magnetic recording. IEEE Trans. Magn..

[2] Childress J.R., Carey M.J., Maat S., Smith N., Fontana R.E., Druist D., Carey K., Katine J.A., Robertson N., Boone T.D. (2008). All-Metal Current-Perpendicular-to-Plane Giant Magnetoresistance Sensors for Narrow-Track Magnetic Recording. IEEE Trans. Magn..

[3] Takagishi M., Yamada K., Iwasaki H., Fuke H.N., Hashimoto S. (2010). Magnetoresistance Ratio and Resistance Area Design of CPP-MR Film for 2–5 Tb/in^2^ Read Sensors. IEEE Trans. Magn..

[4] Hirohata A., Sukegawa H., Yanagihara H., Zutic I., Seki T., Mizukami S., Swaminathan R. (2015). Roadmap for Emerging Materials for Spintronic Device Applications. IEEE Trans. Magn..

[5] Nagamine Y., Maehara H., Tsunekawa K., Djayaprawira D.D., Watanabe N., Yuasa S., Ando K. (2006). Ultralow resistance-area product of 0.4 Ωμm^2^ and high magnetoresistance above 50% in CoFeB/MgO/CoFeB magnetic tunnel junctions. Appl. Phys. Lett..

[6] Maehara H., Nishimura K., Nagamine Y., Tsunekawa K., Seki T., Kubota H., Fukushima A., Yakushiji K., Ando K., Yuasa S. (2011). Tunnel Magnetoresistance above 170% and Resistance–Area Product of 1 Ω(μm)^2^ Attained by In situ Annealing of Ultra-Thin MgO Tunnel Barrier. Appl. Phys. Express.

[7] Butler W.H., Zhang X.G., Schulthess T.C., MacLaren J.M. (2001). Spin-dependent tunneling conductance of Fe|MgO|Fe sandwiches. Phys. Rev. B.

[8] Mathon J., Umerski A. (2001). Theory of tunneling magnetoresistance of an epitaxial Fe/MgO/Fe(001) junction. Phys. Rev. B.

[9] Valet T., Fert A. (1993). Theory of the perpendicular magnetoresistance in magnetic multilayers. Phys. Rev. B.

[10] Felser C., Fecher G., Balke B. (2007). Spintronics: A Challenge for Materials Science and Solid-State Chemistry. Angew. Chem. Int. Ed..

[11] Yakushiji K., Saito K., Mitani S., Takanashi K., Takahashi Y.K., Hono K. (2006). Current-perpendicular-to-plane magnetoresistance in epitaxial Co_2_MnSiCrCo_2_MnSi trilayers. Appl. Phys. Lett..

[12] Iwase T., Sakuraba Y., Bosu S., Saito K., Mitani S., Takanashi K. (2009). Large Interface Spin-Asymmetry and Magnetoresistance in Fully Epitaxial Co_2_MnSi/Ag/Co_2_MnSi Current-Perpendicular-to-Plane Magnetoresistive Devices. Appl. Phys. Express.

[13] Furubayashi T., Kodama K., Sukegawa H., Takahashi Y.K., Inomata K., Hono K. (2008). Current-perpendicular-to-plane giant magnetoresistance in spin-valve structures using epitaxial Co_2_FeAl_0.5_Si_0.5_/Ag/Co_2_FeAl_0.5_Si_0.5_ trilayers. Appl. Phys. Lett..

[14] Nakatani T.M., Furubayashi T., Kasai S., Sukegawa H., Takahashi Y.K., Mitani S., Hono K. (2010). Bulk and interfacial scatterings in current-perpendicular-to-plane giant magnetoresistance with Co_2_Fe(Al_0.5_Si_0.5_) Heusler alloy layers and Ag spacer. Appl. Phys. Lett..

[15] Maat S., Carey M.J., Childress J.R. (2008). Current perpendicular to the plane spin-valves with CoFeGe magnetic layers. Appl. Phys. Lett..

[16] Carey M.J., Maat S., Chandrashekariaih S., Katine J.A., Chen W., York B., Childress J.R. (2011). Co_2_MnGe-based current-perpendicular-to-the-plane giant-magnetoresistance spin-valve sensors for recording head applications. J. Appl. Phys..

[17] Sato J., Oogane M., Naganuma H., Ando Y. (2011). Large Magnetoresistance Effect in Epitaxial Co_2_Fe_0.4_Mn_0.6_Si/ Ag/Co_2_Fe_0.4_Mn_0.6_Si Devices. Appl. Phys. Express.

[18] Sakuraba Y., Ueda M., Miura Y., Sato K., Bosu S., Saito K., Shirai M., Konno T.J., Takanashi K. (2012). Extensive study of giant magnetoresistance properties in half-metallic Co_2_(Fe,Mn)Si-based devices. Appl. Phys. Lett..

[19] Li S., Takahashi Y.K., Furubayashi T., Hono K. (2013). Enhancement of giant magnetoresistance by *L*2_1_ ordering in Co_2_Fe(Ge_0.5_Ga_0.5_) Heusler alloy current-perpendicular-to-plane pseudo spin valves. Appl. Phys. Lett..

[20] Nikolaev K., Kolbo P., Pokhil T., Peng X., Chen Y., Ambrose T., Mryasov O. (2009). “All-Heusler alloy” current-perpendicular-to-plane giant magnetoresistance. Appl. Phys. Lett..

[21] Ko V., Han G., Qiu J., Feng Y.P. (2009). The band structure-matched and highly spin-polarized Co_2_CrZ/Cu_2_CrAl Heusler alloys interface. Appl. Phys. Lett..

[22] Sakuraba Y., Izumi K., Iwase T., Bosu S., Saito K., Takanashi K., Miura Y., Futatsukawa K., Abe K., Shirai M. (2010). Mechanism of large magnetoresistance in Co_2_MnSi/Ag/Co_2_MnSi devices with current perpendicular to the plane. Phys. Rev. B.

[23] Miura Y., Futatsukawa K., Nakajima S., Abe K., Shirai M. (2011). First-principles study of ballistic transport properties in Co_2_MnSi/*X*/Co_2_MnSi(001) (*X* = Ag, Au, Al, V, Cr) trilayers. Phys. Rev. B.

[24] Kubota T., Oogane M., Mizukami S., Naganuma H., Ando Y., Miyazaki T. (2011). Magnetoresistance Effect in Co_2_MnSi/semimetallic-Fe_2_VAl/CoFe Junctions. J. Phys. Conf. Ser..

[25] Li S., Takahashi Y.K., Sakuraba Y., Chen J., Furubayashi T., Mryasov O., Faleev S., Hono K. (2016). Current-perpendicular-to-plane giant magnetoresistive properties in Co_2_Mn(Ge_0.75_Ga_0.25_)/Cu_2_TiAl/Co_2_Mn (Ge_0.75_Ga_0.25_) all-Heusler alloy pseudo spin valve. J. Appl. Phys..

[26] Hase N., Nakatani T., Kasai S., Takahashi Y., Furubayashi T., Hono K. (2012). Effect of NiAl underlayer and spacer on magnetoresistance of current-perpendicular-to-plane spin valves using Co_2_Mn(Ga_0.5_Sn_0.5_) Heusler alloy. J. Magn. Magn. Mater..

[27] Furubayashi T., Takahashi Y.K., Sasaki T.T., Hono K. (2015). Enhancement of current-perpendicular-to-plane giant magnetoresistance in Heusler-alloy based pseudo spin valves by using a CuZn spacer layer. J. Appl. Phys..

[28] Li S., Nakatani T., Masuda K., Sakuraba Y., Xu X., Sasaki T., Tajiri H., Miura Y., Furubayashi T., Hono K. (2018). Enhancement of current-perpendicular-to-plane giant magnetoresistive outputs by improving *B*2-order in polycrystalline Co_2_(Mn_0.6_Fe_0.4_)Ge Heusler alloy films with the insertion of amorphous CoFeBTa underlayer. Acta Mater..

[29] Nakatani T., Mihajlović G., Read J.C., Suk Choi Y., Childress J.R. (2015). High signal output in current-perpendicular- to-the-plane giant magnetoresistance sensors using In-Zn-O-based spacer layers. Appl. Phys. Express.

[30] Balke B., Fecher G.H., Kandpal H.C., Felser C., Kobayashi K., Ikenaga E., Kim J.J., Ueda S. (2006). Properties of the quaternary half-metal-type Heusler alloy Co_2_Mn_1-*x*_Fe_*x*_Si. Phys. Rev. B.

[31] Kubota T., Tsunegi S., Oogane M., Mizukami S., Miyazaki T., Naganuma H., Ando Y. (2009). Half-metallicity and Gilbert damping constant in Co_2_Fe_*x*_Mn_1-*x*_Si Heusler alloys depending on the film composition. Appl. Phys. Lett..

[32] Sakuraba Y., Ueda M., Bosu S., Saito K., Takanashi K. (2014). CPP-GMR study of half-metallic full-Heusler compound Co_2_(Fe,Mn)Si. J. Magn. Soc. Jpn..

[33] Yako H., Sakuraba Y., Amemiya K., Sakamaki M., Kubota T., Sasaki T., Miura Y., Hono K., Takanashi K. Investigation of the exchange stiffness at Co_2_MnSi/Ag and Co_2_FeSi/Ag interfaces using the depth-resolved X-ray magnetic circular dichroism. Proceedings of the 59th Annual Conference on Magnetism and Magnetic Materials.

[34] Kubota T., Ina Y., Tsujikawa M., Morikawa S., Narisawa H., Wen Z., Shirai M., Takanashi K. (2016). Current perpendicular-to-plane giant magnetoresistance devices using half-metallic Co_2_Fe_0.4_Mn_0.6_Si electrodes and a Ag-Mg spacer. J. Phys. D Appl. Phys..

[35] Narisawa H., Kubota T., Takanashi K. (2015). Current perpendicular to film plane type giant magnetoresistance effect using a Ag-Mg spacer and Co_2_Fe_0.4_Mn_0.6_Si Heusler alloy electrodes. Appl. Phys. Express.

[36] Kubota T., Ina Y., Wen Z., Narisawa H., Takanashi K. (2017). Current perpendicular-to-plane giant magnetoresistance using an *L*1_2_ Ag_3_Mg spacer and Co_2_Fe_0.4_Mn_0.6_Si Heusler alloy electrodes: Spacer thickness and annealing temperature dependence. Phys. Rev. Mater..

[37] Jung J.W., Sakuraba Y., Sasaki T.T., Miura Y., Hono K. (2016). Enhancement of magnetoresistance by inserting thin NiAl layers at the interfaces in Co_2_FeGa_0.5_Ge_0.5_/Ag/Co_2_FeGa_0.5_Ge_0.5_ current-perpendicular-to-plane pseudo spin valves. Appl. Phys. Lett..

[38] Jung J.W., Sakuraba Y., Bosu S., Li S., Hono K. (2016). Enhancement of Interfacial Spin-Dependent Scattering of Co_2_Fe(Ga_0.5_Ge_0.5_)/Ag/Co_2_Fe(Ga_0.5_Ge_0.5_) Current-Perpendicular-to-Plane Giant Magnetoresistive Pseudo-Spin Valves. IEEE Trans. Magn..

[39] Aballe L., Rogero C., Horn K. (2002). Quantum-size effects in ultrathin Mg films: Electronic structure and collective excitations. Surf. Sci..

[40] Narisawa H., Kubota T., Takanashi K. (2015). Reply to “Comment on ‘Current perpendicular to film plane type giant magnetoresistance effect using a Ag-Mg spacer and Co_2_Fe_0.4_Mn_0.6_Si Heusler alloy electrodes”. Appl. Phys. Express.

[41] Nakatani T.M., Mitani S., Furubayashi T., Hono K. (2011). Oscillatory antiferromagnetic interlayer exchange coupling in Co_2_Fe(Al_0.5_Si_0.5_)/Ag/Co_2_Fe(Al_0.5_Si_0.5_) films and its application to trilayer magnetoresistive sensor. Appl. Phys. Lett..

[42] Oogane M., Kubota T., Kota Y., Mizukami S., Naganuma H., Sakuma A., Ando Y. (2010). Gilbert magnetic damping constant of epitaxially grown Co-based Heusler alloy thin films. Appl. Phys. Lett..

[43] Oogane M., Wakitani T., Yakata S., Yilgin R., Ando Y., Sakuma A., Miyazaki T. (2006). Magnetic Damping in Ferromagnetic Thin Films. Jpn. J. Appl. Phys..

[44] Slonczewski J. (1996). Current-driven excitation of magnetic multilayers. J. Magn. Magn. Mater..

